# Bad social norms rather than bad believers: examining the role of social norms in bad beliefs

**DOI:** 10.1007/s11229-024-04483-5

**Published:** 2024-02-12

**Authors:** Basil Müller

**Affiliations:** Institute of Philosophy, University of Bern, Bern, Switzerland

**Keywords:** Social norms, Bad beliefs, Epistemic blame, Coordination, Misinformation, Conspiracy-theories

## Abstract

People with bad beliefs — roughly beliefs that conflict with those of the relevant experts and are maintained regardless of counter-evidence — are often cast as bad believers. Such beliefs are seen to be the result of, e.g., motivated or biased cognition and believers are judged to be epistemically irrational and blameworthy in holding them. Here I develop a novel framework to explain why people form bad beliefs. People with bad beliefs follow the social epistemic norms guiding how agents are supposed to form and share beliefs within their respective communities. Beliefs go bad because these norms aren’t reliably knowledge-conducive. In other words, bad beliefs aren’t due to bad believers but due bad social epistemic norms. The framework also unifies different explanations of bad beliefs, is testable and provides distinct interventions to combat such beliefs. The framework also helps to capture the complex and often contextual normative landscape surrounding bad beliefs more adequately. On this picture, it’s primarily groups that are to be blamed for bad beliefs. I also suggest that some individuals will be blameless for forming their beliefs in line with their group’s norms, whereas others won’t be. And I draw attention to the factors that influence blameworthiness-judgements in these contexts.

## Introduction

In this paper, I develop a novel framework to explain why people form bad beliefs. The framework is testable and provides distinct interventions to combat such beliefs. Additionally, it has implications for how we think about the blameworthiness of agents with bad beliefs.

Bad beliefs conflict with those asserted by relevant experts and are maintained regardless of readily available and widely accepted evidence to the contrary. They include belief in conspiracy theories, fake news, or misinformation (Levy, 2021). This notion is broad and somewhat vague (see, e.g., Worsnip, 2022b), but for our purposes, it will do.

There are various explanations of why people hold such beliefs and how we should evaluate their doing so. Some maintain that bad beliefs result from motivated cognition, of agents coming to believe for personal or socio-political gain (see, e.g., Williams, 2019, 2020; Kahan, 2012; Glüer & Wikforss, 2022; Cassam, 2019). Others maintain that such beliefs result from biases allowing for cost-efficient inquiry (e.g., Gigerenzer, 2008), from being susceptible to logical fallacies (e.g., Brotherton & French, 2014) or other reasoning deficits (Pennycook & Rand, 2021; Pennycook et al., 2022). Again, others take these beliefs to stand in relation to social dynamics conducive to cooperation within their group (Blancke, 2023). What such views have in common is that—whilst one might want to grant that agents are practically rational in so believing—there seems to be something epistemically deficient, vicious, and/or blameworthy about how agents with bad beliefs acquire or maintain them (see, e.g., Harris, 2018; Glüer & Wikforss, 2022; Bardon, 2019; Peels, 2023; Williams, 2023; Cassam, 2018). In a slogan: Bad beliefs are due to bad believers. This also has implications for how we are to treat these people. As they are irrational, we might want to educate them (Worsnip, 2022a, p. 44) or simply force them to act as we think is required (Meylan and Schmidt, 2023).

Whilst it’s plausible that these accounts are valid for a subset of people with bad beliefs, I suggest that they might not be fitting for another part of the population. These people aren’t trying to cut corners in their inquiry or form their beliefs for gains in social standing. Instead, they’re doing what they rightly feel they’re supposed to do. In other words, they are following the social epistemic norms guiding how agents are supposed to form and share their beliefs within their communities. Expressions like “Do your own research!” reflect people’s often accurate understanding of the norms that govern their group. People with bad beliefs, much like us—those with supposedly fewer or no bad beliefs—are conformists. We stick to how things are done “around here” and do so in largely reasonable ways. Beliefs go bad because these norms aren’t reliably knowledge-conducive. So, here’s a different slogan: Bad beliefs aren’t due to bad believers but due to bad social norms.

I argue for this by building on existing work on social epistemic norms (e.g., Goldberg, 2018; Greco, 2020; Henderson & Graham, 2017; Simion, 2021) and epistemic styles (Flores, 2021) to establish a framework that allows us to understand bad beliefs as regulation-failures of social epistemic norms. Social epistemic norms are supposed to regulate our epistemic styles, and when they fail to do so properly, bad beliefs result. I submit that the framework has some prior theoretical plausibility in virtue of unifying different explanations of bad beliefs by suggesting a common underlying factor: The misregulation of our epistemic styles by social epistemic norms.

Whilst it’s plausible that many different factors influence people’s forming bad beliefs, I here advocate that social epistemic norms are an important and, relatively speaking, neglected factor. Notably, the notion of “social norms” is at the centre of a well-established, interdisciplinary research field. This allows for the framework to be empirically tested and provides unique interventions to combat bad beliefs—some of which I sample below.

The framework also helps to capture the complex and often contextual normative landscape surrounding bad beliefs more adequately. What one might call the epistemic exonorationists have recently sought to defend people with bad beliefs from allegations of epistemic irrationality. Exonorationists aim to show that the belief-formation of agents with bad beliefs is largely sensible and thus not deserving of blame (e.g., Rini, 2017; Levy, 2021; Meylan and Schmidt, 2023; Begby, 2022; see also Bortolotti, 2022). What follows will agree with them to a considerable degree: I argue that primarily *groups*—rather than individuals—should be blamed for the bad social epistemic norms within them. When it comes to individual blameworthiness, I suggest that *some* individuals truly are blameless in forming their beliefs in line with the norms of their group. However—pace the exonorationists—others plausibly aren’t. In discussing this, I show how our blameworthiness-judgements will depend on the kinds of resources, abilities, and environments agents have at their disposal to make sense of a norm’s reliability, the kinds of reasons available to them to (not) consider others’ social norms and practices, and the overall make-up of their social identities and environments.

## The set-up: epistemic styles and social epistemic norms

### Flores on epistemic styles

The notion of epistemic styles [ES] accounts for systematic differences in how people interact with the available evidence. Flores defines ES as unified approaches to evidence engagement, reflecting a cohesive set of epistemic parameters. Adopting an ES hinges on possessing corresponding dispositions and configuring epistemic parameters accordingly. Differences in how people interact with evidence are thus seen to result from more or less unified dispositions (“epistemic styles”), which express the setting of one or several epistemic parameters.

These parameters^1^ do not only include the amount of evidence that’s required for belief but, more generally, also contain the following:

- how agents value acquiring true vs false beliefs,
- how they value theoretical virtues, such as the number of postulates used to explain a more minor or more extensive set of data,
- the kind of evidence that’s relevant to belief (e.g., statistical evidence vs testimony),
- what kind of sources they take to be authoritative regarding certain questions and/or whose testimony they’ll trust.

Flores discusses both the Rationalist and Black Feminist communities’ styles as examples: Rationalists employ Bayesian principles, embodying a "scout mindset" (Galef 2021) marked by curiosity and open-mindedness. Conversely, Black Feminists—as discussed in Patricia Hill Collins (2002)—prioritize testimony of people with personal experience relevant to the question at hand. Differences in how members of such communities interact with evidence might thus, e.g., be explained by what kind of evidence they take to be relevant to the question at hand and whom they take to be authoritative on specific issues.

ES are meant to differ from and solve some problems of other notions, such as epistemic virtues, in that they don’t ascribe long-standing character traits to agents (Flores, 2021; see for an overview of virtue epistemology Turri et al., 2021). One key aspect of epistemic styles is that they’re context-sensitive: Depending on the context, agents’ epistemic parameters will be set differently, and so the resulting styles will also differ. This makes the notion ideally suited for what’s to come below, as I’ll argue that one contextual factor—the presence (or absence) of social epistemic norms—influences ES.^2^

Going beyond Flores, I posit that ES have an epistemic function, namely to promote the acquisition of epistemic goods (such as knowledge, true beliefs, or understanding) within the agent’s respective environment and overall context (see, e.g., Simion, 2019; Milikan, 1984; Burge, 2010; Graham, 2012 for similar accounts). To foreshadow a bit, this will prove useful when considering how we should evaluate ES in Sect. 5.

We can individuate ES by how they fulfil this function: different ES will attain epistemic goods in different ways. The reason why there are different ES plausibly lies in the fact that agents are part of different communities with different histories and environments that have different epistemic needs and affordances. Agents themselves will also have different needs and abilities regarding their epistemic lives. For example, the Rationalist and Black Feminists styles differ in how they fulfil the function of acquiring epistemic goods, and the reason for this is that their communities are differently constituted—both historically, but also culturally and with regards to the make-up of their members.

Having grasped the general framework, an interesting question arises that Flores doesn’t address: Which factors impact agents adopting a particular ES? The answer to this is likely to be both complex and important: It’ll be complex because it’ll likely refer to psychological, sociological, and potentially historical factors. Whilst the notion of ES does allow for a degree of agency in how parameters are being set (Flores, 2021, p. 36),^3^ it’s plausible that they aren’t set voluntarily and, as already mentioned, impacted by a host of contextual factors. It’s important for the same reason that the notion itself is important: It’ll allow us to understand and rationally engage with others—and, as I’ll argue below, it’ll allow us to understand bad beliefs.

### Social epistemic norms: between cooperation and coordination

I suggest the answer to why people engage in an ES will significantly involve social epistemic norms [SEN]. By “significantly involve”, I mean to acknowledge that other factors will also contribute to why people engage in an ES. So, whilst SEN won’t suffice to provide a comprehensive account, they’ll nonetheless prove to be informative.

Social norms are rules that guide agents in which behaviours are appropriate, permissible, or obligatory in specific situations and communities. More specifically, the notion of “social norm” used here is inspired by game-theoretic accounts, where social norms are seen to solve collective action problems (Young 1998; Gintis 2014; Lewis 1969). On this view, social norms function to regulate collective action so that public goods and/or shared goals can be attained.

SEN are norms that guide agents regarding their inquiry and in how they are to form and share their beliefs. They might have explicit epistemic content, such as “If you’d like to find out whether p, only trust the opinions of these experts regarding p.” Or they might have indirect implications for epistemic conduct, such as, e.g., “Respect your elders”, or “Be loyal to your friends!” They operate in epistemically interdependent social groups and allow for the attainment of epistemic goods, such as true beliefs, knowledge, or understanding. Within such groups, agents depend on each other or need to collaborate in various ways to attain epistemic goods (see, e.g., Graham, 2015; Greco, 2020; Goldberg, 2018; Henderson & Graham, 2017; Simion, 2021).

We can distinguish between two kinds of SEN. Doing so will be important, as understanding why bad beliefs arise requires us to understand the failure modes that different SEN have.

First is what I will call [cooperation-norms] (Simion, 2021; Handfield, 2023; Greco, 2020; Goldberg, 2018; Graham, 2015; Faulkner, 2011; Henderson, 2020). Cooperation-norms stabilise cooperation against the threat of defection, cheating, or free-riding (Fehr & Schurtenberger, 2018; Fehr et al., 2002; Gintis et al., 2008). In the empirical literature, they’re sometimes called injunctive norms (due to Cialdini et al., 1990; Reno et al., 1993; see also Legros & Cislaghi, 2020). The general idea is that agents might be inclined to promote interests or aims that go against those of their social group, thus hindering the attainment of public goods. For the epistemic case, agents might be incentivised to deceive others or form beliefs for non-truth-related reasons and thus impede the attainment of true beliefs in the epistemically interdependent social groups they’re a part of (Handfield, 2023; Henderson, 2020; Müller, 2022).^4^ Cooperation-norms change the payoff-structure of these interactions such that it's more advantageous for individuals to behave in the required ways. The relevant mechanisms here are usually seen to be sanctioning, reputation, and internalisation (Henrich & Muthukrishna, 2021; Graham, 2015).

However, SEN do more than just deter lazy believers and would-be deceivers. They also coordinate and inform the epistemic behaviours of earnest epistemic agents. I call this kind of norm a [coordination-norm]. They’re similar to what’s often called descriptive norms (e.g., Bicchieri, 2016, Chap. 1; Legros & Cislaghi, 2020; Reno et al., 1993). Coordination-norms emerge in contexts where there are multiple different ways in which agents could act to attain the good in question; and where it’s unclear which option they should choose. There’s an important motivational difference to cooperation-norms: Agents needn’t be incentivised to act in the ways required of them—they’re already interested in attaining the good—it’s rather that it’s unclear how they should go about doing so. Coordination-norms facilitate this by providing standards for the respective behaviours. The result is coordinated behaviour across the group.

Some coordination-norms will be conventions: Conventions arise in cases where there are, at least in principle, equally productive ways of attaining a good, yet there’s a need for agents to behave in coordinated ways for this to succeed. Think of the norms that tell us on which side of the road we’re supposed to drive on. Some SEN will be conventions. Epistemic conventionalists, for example, maintain that which reasoning-style we’re supposed to employ is a matter of convention (Carr, 2022; Dogramaci, 2012, 2015).

Not all cooperation-norms are conventions, however. Coordination-norms also promote an agent’s need to acquire information about how the good is to be attained when individual learning isn’t practical. Consider the norms of fashion: Unfashionable agents can turn to agents whose way of dressing reflects the relevant standards of fashionableness. In the below, I suggest that, like cooperation-norms, conventions and fashion-styles, agents look to SEN to adjust their ES.

### Epistemic styles and social epistemic norms

Social epistemic norms influence epistemic styles by influencing how the underlying epistemic parameters are set. I’ll call the resulting framework the epistemic styles and social epistemic norms framework or the [ESSEN-framework]. Importantly, this is, at this point, a descriptive rather than a normative framework: It’s supposed to accurately explain how SEN bring about bad beliefs. I’ll turn to normative matters in Sect. 5.

Cooperation-norms incentivise agents to adhere to a particular epistemic style by incentivising them to set their parameters such that shared epistemic goods can be attained—even if, in the absence of such a norm, they might prefer to set them differently. Epistemic cooperation-norms might, for example, guide scientists to uphold relatively high evidential thresholds regarding their statistical analysis, even if lower ones would be more advantageous regarding their ability to publish flashy findings.

Coordination-norms coordinate how agents set their parameters to attain epistemic goods. Again, this isn’t a matter of motivation/incentivisation but instead of informing agents on how they should behave to attain epistemic goods. Sometimes, coordination-norms will instruct agents how to set their parameters in relation to each other such that they can collectively attain epistemic goods. In other instances, such norms might instruct agents how to set their parameters solely because it’s unclear how to do so. For example, coordination-norms might instruct two collaborating research-groups how they’re supposed to set their epistemic parameters in relation to each other—e.g., how much and the kind of evidence that’s relevant to support the hypothesis—such that their collaboration succeeds. Or they might instruct agents curious but clueless about a question to whom they should defer to.

Before going into more detail regarding how the ESSEN-framework captures bad beliefs, I’d like to acknowledge that the causal relationship between SEN and ES is likely to be complex. Plausibly, it’s not just the case that SEN influence ES but that the presence of specific ES within a community, especially amongst highly visible or influential agents, will lead to particular SEN to be established and maintained. As such, the causal relationship between SEN and ES might be best described as bi-directional, at least on the level of social groups: SEN influence agents’ ES, but the kinds of ES we find in social groups also influence the kinds of SEN that can emerge and stabilise.^5^

I take it that there are two different—albeit closely related—explanatory projects here: One is about how SEN influence ES and the other is about how SEN emerge and what role ES play there. My main aim here is to lay the groundwork and draw attention to how bad SEN influence agents’ ES and lead to bad beliefs. Once we’ve understood this, it seems like a natural next step to ask how bad SEN emerge and how they could be changed. I advocate for subsequent research to consider these questions more closely and will again touch on them in Sect. 4.

In summary, the ESSEN-framework proposes that SEN lead agents to employ specific ES by influencing how the underlying epistemic parameters are to be set. The following reasons speak in favour of the ESSEN-framework: First, as Flores points out, we can observe uniformity in ES within particular social groups (think of the Rationalists and Black Feminists examples). This is well explained by agents adhering to shared SEN. Second, non-epistemic styles, e.g., in fashion, are similarly influenced by social norms (see, e.g., Bicchieri, 2005 on fashions and fads). Third, our reactive attitudes also reveal the existence of norms: We’d praise or blame agents for (not) employing the correct ES (Boult, 2021; Dogramaci, 2012), we’d hold them responsible for doing so (Meylan, 2017), or, at the very least, we’d explain to them how things are done around here (e.g., we trust journalist x over y). Fourth, our judgement of the ESSEN-framework’s plausibility ultimately comes down to its explanatory power. Below, I argue that it not only helps us understand bad beliefs but is also open to empirical investigation and provides unique interventions to combat such beliefs.

## The Essen-framework and bad beliefs

How is the ESSEN-framework supposed to help explain bad beliefs? The general idea is this: Bad beliefs are instances of norm regulation failures. In cases of bad beliefs SEN fail to fulfil their function by not regulating ES properly. As SEN regulate collective epistemic undertakings so that epistemic goods can be attained, more or less widespread and systematic instances of bad beliefs constitute a failure of such norms to perform their function.^6^

Since cooperation- and coordination-norms fulfil different functions, distinct kinds of regulation-failures come with them. Recall cooperation-norms regulate collective action so that agents don’t cheat or free-ride; coordination-norms do so by allowing agents to coordinate their behaviour and by informing ignorant agents.

For cooperation-norms, a regulation-failure might result from the insufficient strength of the incentives or what they’re being targeted at (Müller, 2022). Whereas in the former case diverging from the norm might not be costly enough due to a relative lack of the strength of sanctions, in the latter case, even very strong sanctions might be inefficient because they’re aimed at the wrong behaviour. For example, sanctioning deception is futile if the problem lies in belief-*formation*. Cooperation-norms might also fail because they actively incentivise agents to display the wrong kind of doxastic behaviour, e.g., if they incentivise agents to believe in line with the group’s dominant-yet-false ideology.

For coordination-norms, regulation-failures can occur because the target behaviours aren’t well-coordinated, meaning behaviour A and behaviour B will, in concert, not be conducive to attaining collective epistemic goods. This can occur within and between groups, as the research-group case above indicates. Coordination-failures might also occur when epistemic norms instruct agents to set their parameters in ways that aren’t productive in the first place. Here, bad beliefs result because an agent’s epistemic parameter wasn’t set productively—they might, e.g., defer to the wrong agents.

Ultimately, the degree to which norm-regulation failures are responsible for bad beliefs is an empirical question (see Sect. 4 for predictions of the ESSEN-framework and its testability). However, the framework does have some prior theoretical plausibility. Below I sample a variety of competing explanations of bad beliefs and suggest that they are unified by a common underlying factor: The misregulation of ES by SEN. The framework thus provides a unified perspective on a dispersed set of explanations of bad beliefs. As we’ll see, though the role of SEN isn’t made explicit, many of these accounts fit well with the framework. In other instances, the framework allows us to reinterpret the account’s findings. This will also exemplify the kinds of bad SEN that lead to bad ES (and the corresponding bad beliefs), thus developing the view in more detail.

In discussing these accounts, the following pattern will emerge: The accounts will describe different epistemic behaviours that lead to bad beliefs. In other words, the explanations I sample will refer to different (aspects of agents’) epistemic styles and to the settings of different epistemic parameters that are responsible for bad beliefs. In turn, I’ll highlight how these explanations can be gainfully re-interpreted such that SEN are plausibly responsible for the epistemic behaviours that are being discussed. The suggestion will be that the ES these explanations refer to are the product of agents conforming to the SEN of their communities.

Levy (2021) suggests that agents need to outsource the evaluation of first-order evidence regarding most questions they care about due to the issues of scope and complexity and constraints on time and cognitive resources. The difference between the agent who holds bad beliefs and the one who does to a lesser degree isn’t one of cognitive mechanisms—both defer by considering the available higher-order evidence—but rather a difference in environment. Whereas the former inhabits an epistemic environment with polluted first- but especially higher-order evidence, the latter do less so.

In line with this, Levy suggests that fixing the epistemic environment is a collective action problem, “made worse by the fact that some actors don’t share the goal most of us would like to achieve” (Levy, 2021, p. 127). Because of these actors, “Reducing epistemic pollution will almost certainly require some degree of coercion, from government or other institutions with the clout to impose costs on those who don’t cooperate.” (Levy, 2021, p. 127).

Viewed from the vantage point of the ESSEN-framework, we can see that bad SEN are part of agents’ socio-epistemic environment and are thus one factor that contributes to its pollution. They might, for example, instruct them to defer to non-mainstream sources or guide them regarding the true markers of expertise. As a result, agents’ ES will be sensitive to certain instances of higher-order evidence—evidence due to non-mainstream sources or from agents possessing supposed markers of expertise—but not to others.

It's important to note that whom one defers to is subject to SEN: Interdependent social groups wouldn’t function if agents deferred to different agents. For example, they’d be unable to trust each other’s testimony, for they might have gotten it from sources they themselves deem untrustworthy (Dogramaci, 2015). Additionally, as Levy argues, figuring out whom to defer to—especially regarding complex issues—is a daunting task for any individual agents. This is why we rely on social structures and practices to identify whom we should trust (Levy, 2021, Chaps. 4 & 5). SEN are part of these social practices. They regulate agents’ ES by influencing whom agents defer to, i.e., by influencing how the epistemic parameters concerned with whom agents deferred to are set.

Notably, this picture fits well with Levy’s take: He agrees that there’s a sense in which bad beliefs result from a failure in collective action. On the one hand, Levy maintains that a subset of agents doesn’t seek to promote epistemic aims—this is why the epistemic environment is polluted. These agents likely would need to be coerced into doing so. This then is a clear instance of a regulation failure of a cooperation-norm: The cooperation-norms operative in these communities either didn’t sufficiently incentivise agents to display the correct epistemic behaviour or did incentivise them to display behaviour that led to the pollution of the epistemic environment. In the former case, agents might, for example, have not been sufficiently incentivised to be truthful regarding their status as an expert. In contrast, agents might have been incentivised to make misleading claims to promote their institution’s financial aims in the latter. Either way, bad SEN lead to agents polluting the environment with misleading first- or higher-order evidence.

On the other hand, many agents with bad beliefs are solely the subject of unfortunate circumstances on Levy’s view: Because their first- and second-order evidence is polluted, they end up with bad beliefs by deferring in ways not conducive to their epistemic aims. Here a failure of coordination-norms looms large, as these agents did seek to acquire epistemic goods, presumably by conforming to such norms. However, rather than allowing them to successfully coordinate with others or gain accurate information regarding how they should set their epistemic parameters, these norms guided agents to defer to the wrong sorts of individuals and institutions.

Rini (2017) maintains that some bad beliefs are due to an ambiguity in the norms about sharing content online: It’s unclear whether sharing—e.g., through retweets—entails assertion. Importantly, people’s default when encountering shared content, especially if the same content is shared multiple times, seems to be to accept it as true. In light of this, Rini advocates disambiguating these norms to combat bad beliefs.

Again, we can understand Rini’s account to support the ESSEN-framework. Here too bad beliefs result from a failure of SEN. On one reading, the lack of norms guiding agents in what they can take shared content to entail is causing bad beliefs. We can see that there’s a need for such norms: Interdependent agents can’t successfully collaborate if they understand evidence differently. They’ll neither trust the other’s reasoning nor comprehend where they’re coming from. Additionally, how to interpret others’ behaviour online—especially if one doesn’t know them—is difficult to do correctly. As Rini rightly notes, this is why we rely on SEN to provide shared standards. As a result of this lack of SEN, agents’ ES were not well-coordinated: They took the content others shared online to be asserted when it wasn’t meant as such.

There are two important differences to Levy: First, whereas Levy maintained that social groups coordinated on the wrong agents to learn from, Rini maintains that they failed to coordinate at all. Perhaps more importantly, Levy and Rini appeal to different epistemic parameters that fail to be well-regulated: Levy seems to focus on the source of testimony—whose first- and higher-order evidence we should rely on. In contrast, Rini focuses on what we can take certain forms of expression to mean—i.e., whether sharing that p online implies asserting that p. Whilst one is about who is providing the evidence, the other is about what we can draw from the kind of evidence that is being provided.

On another reading of the cases Rini discusses, it’s not the lack of norms that’s responsible for bad beliefs, but rather the presence of bad SEN.^7^ Plausibly, in the cases that she describes, there might have been a toxic combination of norms that instructed agents to i) share content if it’s interesting and ii) take content that is shared to be asserted. Here, the norm-regulation failure would be due to these norms not being epistemically productive when applied in concert. There’s preliminary evidence that this does occur: Altay et al. (2022) present evidence that people’s sharing of news online (both fake and actual) was driven not only by accuracy but also by how interesting the content was if it were true. On the reading I propose, this kind of behaviour is evidence of and resulting from agents conforming to the SEN of their communities.

Fraser (2020), in her review of Cassam (2019) and Rosenblum and Muirhead (2019), suggests that “Contemporary conspiracism [a prime example of bad beliefs] is a coupling of Cartesian paranoia with a very unCartesian passional structure: epistemic fear of missing out, or FOMO.”^8^

Conspiracy believers are said to not only be sceptical that the evidence indeed favours what it appears to—the familiar “Cartesian Paranoia”—they also have an extreme preference for acquiring true beliefs, even if this means that false beliefs will be acquired as well—epistemic FOMO. If what I fear most is to miss out on true beliefs, then simply believing on strong evidence won’t do, as this leads to missing out on the true beliefs for which one’s evidence might not be as strong. Fraser maintains—similar to James (1979)—that, at least in principle, there’s nothing epistemically wrong with having such a preference-ordering. The problem instead lies in this way of believing not being suited to establishing the shared sense of reality that democracy and public debate require.

We can see that here, too, a norm regulation-failure is plausible. Agents’ epistemic behaviour can be (partially) explained by invoking SEN: Agents’ ES were influenced by SEN regarding the acquisition of true beliefs over avoiding false ones. These norms might have instructed agents to never miss out on the truth, not to trust that things are as they appear, or to take seriously even the most minor bits of evidence that disconfirm the “official story”. On my proposal, these SEN influenced agents’ ES, resulting in exactly the mixture between Cartesian Paranoia and epistemic FOMO that Fraser describes.

How agents are to set this particular parameter is subject to SEN, as successful collective inquiry requires that agents are equally risk-seeking when forming beliefs. If one agent believes on very little evidence and the other does not, it’d be hard for either of them to trust the other’s assertions. Whereas preferring to not miss out on true belief on the cost of acquiring false ones might serve agents within a particular social group reasonably well, coordination between different groups and society at large becomes increasingly difficult, as most social groups prefer not to have false beliefs rather than acquiring true ones (Altay, 2022; Jahanbakhsh et al., 2021).

Grundmann (2021) offers yet another account that can be read to support the ESSEN-framework. Alongside others (e.g., Buzzell & Rini, 2022; Levy, 2022; Matheson, 2022), he believes the Enlightenment principle of critical thinking is essential in explaining bad beliefs. Roughly put, such principles maintain that when considering whether p, one must always consider one’s own reasons regarding p.

Grundmann maintains that people come to have bad beliefs by relying on their reasoning capacities in the spirit of these Enlightenment principles. Agents perform plausibility-checks regarding the theories held by the relevant experts: They ask whether their assertions are plausible in light of their own reasons and reasoning. This leads to bad beliefs because “true scientific and expert views often are so radically different from laypeople’s background beliefs that they must look outrageous to them.”(Grundmann, 2021, p. 147).

The key point here is that these Enlightenment principles are SEN. They tell agents to make use of their own reasons and reasoning, or how it’s these days rather often put: “Do your own research!” The resulting ES are overly epistemically individualistic: Agents tend to think for themselves rather than defer to others—just as the norm prescribes. Thus, we can understand these agents’ epistemic behaviours as regulation failures of such norms. They instructed agents to think for themselves when they really shouldn’t have. In particular, these social groups failed to coordinate on *when* agents should think for themselves and *when* they should defer. When agents should learn from and rely on others and when they should think for themselves is a delicate—because often contextual—balance for social groups to strike. Not only should this balance be struck differently for different belief-contents and believers, but there’s also an additional trade-off to manage:

- On the one hand, individual learning is faulty and often leads to a loss of knowledge, as demonstrated by Grundmann. In interdependent social groups, this can have consequences far beyond the individual. More generally, because of the scope and complexity of knowledge, agents need to depend on others and learn socially.
- On the other hand, solely learning from others isn’t a productive strategy since no new knowledge would enter the group (Hoppitt & Laland, 2013). Additionally, individual learning might build up relevant epistemic capacities over time (Bland, 2022) or be conducive to acquiring other epistemic values such as understanding (Levy, 2022; Matheson, 2022).

So, social groups must regulate when agents think for themselves and when they should defer. Regulation-failures will lead to false or insufficient amounts of information to enter the group and thus for epistemic aims to go unattained. It’s well documented that agents are guided by rules detailing when to learn from others (and from whom)(what’s often called selective social learning, for an overview Heyes, 2018, Chap. 5; Hoppitt & Laland, 2013). The suggestion here is that these rules are SEN, and that (some) bad beliefs are due to their failures to regulate agents’ ES properly.

Lastly, research on identity protective cognition [IPC] describes the tendency of individuals to sample and process information with the aim of protecting or enabling their status as a member of a desirable social group (see, e.g., Kahan, 2012, 2017; Kahan et al., 2012; Petersen et al., 2013; Van Bavel and Pereira 2018; see Pennycook & Rand, 2021 for discussion). On this picture, bad beliefs result from agents seeking to protect their political identity over attaining true beliefs, e.g., by (i) only considering a subset of the available evidence, (ii) by not looking for new evidence, (iii) by selectively recalling evidence supporting the desired belief, and, more generally, (iv) by only investing a certain amount of resources into inquiring and thinking about the relevant proposition (see, e,g., Williams, 2020). IPC is thus an instance of motivated cognition, a phenomenon where our motivations—e.g., our desires, aims, wants, and goals—can causally influence how we form beliefs (see, e.g., Bénabou & Tirole, 2016; Kahan et al., 2012; Kunda, 1990). This is one of the most prominent explanations of bad beliefs in the empirical literature (but see, e.g., Pennycook & Rand, 2019 for criticism).

The ESSEN-framework affords a more complex picture. First, there’s an alternative diagnosis: Agents don’t seek to protect their political identity but instead follow the norms of their community. Such norms might, e.g., tell agents that, when it comes to topic x (say who killed JFK), *this* is the relevant evidence (e.g., the evidence discussed on 4Chan). There’s a need for social norms here, as evaluating which evidence is relevant to any given belief and when one ought to look for new evidence is often less clear than one might assume, especially regarding the complex topics that bad beliefs are about. The reason for this is two-fold: (i) Evaluating what evidence is relevant to an expertise requiring question itself requires expertise. The same goes for when to look for new evidence, as this requires evaluating when the existing evidence isn’t sufficient. Additionally, (ii) due to the sheer extent and fast-changing nature of the available evidence, evaluating it involves a lot of time and resources—resources that agents don’t necessarily have access to. This is why we outsource these things to others. And when it comes to political questions, we outsource to political parties and communities. It’s the norms of these communities then that fail to regulate the ES of agents correctly by incentivising them to consider certain instances of evidence and ignoring others.

Second, the ESSEN-framework also allows for a more complex picture in the sense that SEN plausibly interact with the motivations and needs of individuals and their communities in nuanced ways. Consider that SEN that protect a group’s political identity might find uptake more easily within that group. For example, a norm that specifies a set of relevant and a set of irrelevant instances of evidence might find uptake in a group more easily if the evidence is conducive to retaining the group’s political identity. Much in the same way, individuals might conform to certain SEN more readily precisely because doing so allows them to protect their beliefs. Lastly, it might also be the case that the SEN of particular communities prescribe agents to keep hold of cherished beliefs and sacred convictions in spite of what appears to be counter-evidence (see Williams (2023) for a picture that also alludes to social factors in motivated cognition).

Evaluating which of these options is accurate goes beyond the scope of this paper. The suggestion here is merely that SEN do plausibly play a part in these phenomena and, consequently, that future empirical research should consider their role in more detail. Real-world cases will likely involve many factors: misleading evidence, norms that prime agents to consider that evidence, and non-epistemic reasons that lead agents and groups to employ such norms.^9^

In summary, we’ve seen that different explanations of bad beliefs can be unified by positing regulation-failures of SEN as a common underlying factor. But although these explanations are unified in this way, they differ regarding the epistemic parameters they take to have been misregulated. Agents’ ES were misregulated by SEN with regard to: (i) whom they could trust or consider to have expertise, (ii) what they could take content that’s shared online to imply, or when they should share things online, (iii) when they are supposed to defer to others or when they are supposed to think for themselves, (iv) how they should value the acquisition of true vs. the avoidance of false beliefs, and (v) what kind of evidence they should consider, what they can safely ignore, and when to look for new evidence.

As a result, agents employed deleterious ES, as part of which they placed their trust in agents not deserving of it, were overly credulous when encountering information online (or shared things to freely themselves), were intellectually independent when they should have been deferential, were overly epistemically risky, and ignorant of evidence relevant to their questions. I take it to be a strength of the ESSEN-framework that it’s general enough to encompass these different—and plausibly interrelated—aspects of how agents come to hold bad beliefs.

Let me clarify how the ESSEN-framework relates to the above theories. It complements, rather than competes with, the explanations that some of these accounts provide. For example, I don’t deny that agents attain bad beliefs because they prefer to i) acquire true beliefs over avoiding false ones (Fraser) or ii) over-rely on their own reasoning (Grundmann). The framework adds to these explanations by drawing attention to what brings about this kind of behaviour: It’s because of bad SEN in these communities that agents attain these specific ES and the bad beliefs that result from them. Relatedly, I don’t deny that agents form bad beliefs due to a polluted epistemic environment, where they’ll be exposed to misleading or unreliable first or higher-order evidence, as Levy suggests, or where it’s unclear whether people assert what they share online, as Rini thinks. However, these theories have not gone into great detail concerning which aspects of the epistemic environment they take to be polluted. Notice here that SEN are part of the agent’s epistemic environment. What I’m doing here is drawing attention to a particular aspect of an agent’s epistemic environment—it being structured by the presence or absence of SEN—as an important factor in explaining why agents behave as they do. It’s these norms that are (at least partly) responsible for agents taking certain kinds of higher-order evidence as relevant to whether they should believe that p or for them to share or come to believe things online. So, the proposal here is that a particular environmental factor—SEN—influences ES to produce the kinds of behaviours that the above accounts describe.

The framework also complements existing accounts by allowing them to be more nuanced. The discussion of IPC is illustrative here, as the framework complicates how we think about such cases: On the one hand, there’s a competing explanation of the findings of IPC—and, presumably, other theories that posit individual-level irrationality. Agents themselves aren’t trying to protect their political identity; they’re much rather following the norms of their communities. But, on the other hand, as the discussion above revealed, it might also be that SEN that protect a group’s identity are taken up more easily or that these SEN themselves prescribe to uphold cherished beliefs. What emerges is thus a more nuanced picture of how bad beliefs emerge, a picture that’s deserving of additional investigation.

More generally, the framework differs from all the above theories in providing a more unified perspective on a dispersed set of explanations of bad beliefs by suggesting a common underlying factor: the presence or absence of bad SEN. As we’ll see in the next section, it also differs from them by providing unique predictions regarding how bad beliefs can be changed.

## The ESSEN-framework’s implications for how bad beliefs can be changed

### The ESSEN-framework’s predictions

The unified perspective the framework provides matters because it allows for distinct and testable predictions regarding how we can change bad beliefs and the epistemic styles that cause them. Suppose we think that it’s normatively desirable for a group with bad beliefs to change their epistemic styles. How would we go about achieving this? On a picture that denies the influence of social norms, one might think that what needs to be changed is the agents’ personal epistemic preferences. Such a theory of change maintains that these agents—for whatever reasons—prefer a particular ES, and this preference is the root of bad beliefs. An agent might, for example, prefer to trust specific agents over others, conserve their political identity over acquiring truth, or value acquiring true beliefs over avoiding false ones too strongly. Much as in fashion, some agents have a bad sense of style—they’ll dress or believe in ways that aren’t conducive to acquiring aesthetic or epistemic goods.

To be clear, such theories wouldn’t need to give up on claiming that agents have these preferences because of individual-level irrationality, a polluted environment, or negative social influences. Just as one might dress badly because other members of one’s group also dress this way; one might come to believe badly for the same reasons. The point here is that agents’ personal preferences are shaped by those around them and their environment—and it’s these personal preferences regarding their ES that lead to bad beliefs.

In consequence, one might think that we’d solely need to change agents’ personal references regarding how they value acquiring true over avoiding false beliefs, whom they trust and defer to, whether they value their own political or group-identity over truth, or what they take shared content to mean. As long as these agents would develop the right kinds of personal preferences regarding their ES (and would adapt them accordingly), we’d avoid bad beliefs. As a result, we might seek to change their style by convincing them they’re not productive. We might convince these agents that they should trust agent x over y, that truth is more important than group-membership, that they shouldn’t believe what they see online, etc. If they personally come to accept this, then—so the idea goes—they’ll change their ES, and all will be well.

This prediction will not pan out if SEN influence ES as I’ve described. The reason for this is as follows: If social norms regulate a behaviour, our personal preferences—though certainly important—aren’t sufficient to induce change in that behaviour (see, e.g., Constantino et al., 2022; Bicchieri et al., 2023; Bicchieri, 2016). Agents don’t solely display such behaviours because of their personal preferences but because doing so allows them to coordinate and cooperate with others to attain important goods. Just as in fashion, we don’t just believe (or dress) as we do because of our own personal preferences—because that’s what we deep down think is the best way to dress or believe. Instead, we dress or believe as we do because we’d like to relate to others in specific ways—because we’d like to and need to be part of specific communities.

In other words, agents’ personal preferences aren’t sufficient to induce change because they participate in collective action. Successful collective action requires more than agents having the right personal preferences. Why is that? Consider that agents depend on other members of their social group to acquire goods that are of importance to them. As such, whether it makes sense for these agents to change their behaviour depends not only on their own preferences but on what others do, believe, and expect of them. For example, agents might be sanctioned if they violate their group's norms. But, more importantly, they won’t be able to coordinate and cooperate with others and won’t, therefore, be able to attain the goods they require. So even though an agent themselves might prefer to Φ, because Φ-ing wouldn’t allow them to coordinate or cooperate with others, it won’t make sense for them to Φ. So, agents need not only personally prefer to Φ, but they must also prefer that others Φ, and they need to be reasonably certain that others have similar preferences and expectations.

This is supported by empirical research demonstrating that change in personal preferences isn’t sufficient to bring about behavioural change in cases where that behaviour is subject to social norms. Examples include child marriage and female genital mutilation (see, e.g., Bicchieri, 2016; Constantino et al., 2022): Even if parents might have a personal preference for not mutilating their child, the social system they’re a part of—and on which they and their family depend in many ways—pressures them to do so. As such, simply changing their preferences isn’t sufficient to induce behavioural change.

For the epistemic case, agents would be unable to participate in their group’s collective inquiry. Thus, they would rightly fear missing out on acquiring epistemic goods, as agents must depend on their community to acquire them in many contexts. In addition, given their bad reputation as epistemic agents, it’s likely that these agents would be unable to participate in other cooperative ventures and lose out on other goods. For example, let’s imagine an agent A that would prefer to defer to X rather than Y—say because they’ve been convinced that X is more reliable than Y. If the agents that A depends on, those A would like to collaborate with, do defer to Y, then A won’t thereby come to change their ES so that they defer to X. The reason for this is that collective inquiry requires agents to defer to those that others trust—A wouldn’t be trusted or taken seriously if they’d defer to X and thus would be precluded from seeking to acquire epistemic goods with that particular group.

So, in summary, the ESSEN-framework predicts that simply changing personal preferences won’t suffice to change bad beliefs. Luckily, the notion of “social norm” is at the centre of a well-established and mature interdisciplinary research field. There’s extensive literature on measuring the involvement of social norms in different behaviours (see, e.g., Bicchieri et al., 2023; Legros & Cislaghi, 2020; Constantino et al., 2022). Applying both quantitative and qualitative methods, we could not only test the above prediction but also better understand whether the norms operative in bad beliefs are cooperation- or coordination norms, what their specific content is, which epistemic parameters they influence, and for which reasons agents conform to these norms.

### How to change bad SEN?

But if changing agents’ personal preferences regarding their ES isn’t sufficient, what must happen so that agents don’t form bad beliefs? While no straightforward solution is available, an extensive literature exists on how the social norms operative in a community can be changed (Bicchieri, 2016, Chaps. 3 & 4; again, see Legros & Cislaghi, 2020; Constantino et al., 2022 for overviews). Some of these insights have already been applied to change bad beliefs (e.g., Ecker et al., 2022; Gimpel et al., 2021). Whereas it’s beyond the scope of this paper to provide a full account of how SEN-change might happen, let me highlight two points here:

First, different kinds of social norms call for different types of interventions to be changed. For example, it’s thought that coordination-norms can be altered by providing information about people’s actual behaviour—e.g., for bad beliefs: whom agents actually defer to, when they actually think for themselves, etc. In contrast, a change in cooperation-norms will likely involve a change in the associated sanctions, amongst other things (Centola, 2018; Constantino et al., 2022; Legros & Cislaghi, 2020).

Second, the framework assumes that SEN influence ES. However, as described above, things will also work out the other way around: Agents’ ES influence SEN. Just like fashion-norms are influenced by people’s styles, so too are the norms governing ES: Plausibly, the prevalence of influential people displaying a certain ES will lead to it being picked up by others, eventually establishing itself as a norm. Research on social norm interventions is useful for employing this insight to change social norms for the better. One prominently discussed way in which social-norm-change can be achieved is through tipping-point strategies (see, e.g., Winkelmann et al., 2022; Ginkel et al., 2020; Young, 2015). A tipping point describes the moment when a sufficiently large subset of the population—though still a minority—prefers conforming to a new norm. Once such a threshold is reached, this can set off cascading effects, where more and more agents will conform to the new norm because doing so will allow them to coordinate with others. For the epistemic case, the idea is that people adopt new ES because a sufficient subset of people have spearheaded them.

Now, the question becomes how such tipping-points can be engineered. Whereas it’s traditionally been thought that high-status individuals would be effective sources of norm change—qua being well-connected and influential (Dannals & Miller, 2017; Granovetter, 1973)—more recent research suggests that such a strategy isn’t as promising after all. Why? High-status individuals are less willing to take up counter-normative positions. After all, how their social group is organised does work rather well for them. Current research instead suggests focusing on individuals located more at the periphery of their group. These individuals often have “wide bridges”—i.e., connections to multiple social groups. Such agents come into contact with various ES and aren’t as incentivised to conform to the dominant ones. Constantino et al., (2022, p. 63) mention that this idea “is supported by analyses of the Black Lives Matter movement, the Me Too movement, and support for marriage equality on social media (Centola, 2021b; Kanter, 1993/2008; State & Adamic, 2015)”, where agents more towards the fringes of their social groups contributed to more just norms. Figuring out how to empower agents with both wide bridges and productive ES to drive norm change thus seems like a sensible way forward.

### What evidence would speak against the ESSEN-framework?

Having discussed a prediction unique to the ESSEN-framework, let me briefly acknowledge the kind of evidence that would speak against the influence of SEN on ES.

First, we might find that agents have bad beliefs on some topics but not others. At least at first sight, this would suggest that belief-contents rather than SEN lead to bad beliefs. Agents might feel strongly about certain topics and would like to protect the related beliefs. So, an explanation invoking motivated cognition seems more apt than one that invokes SEN.^10^ While acknowledging this possibility, I’d like to highlight two points to qualify this somewhat: i) SEN are often context and in particular, content-sensitive. SEN not only generally apply to specific agents in particular contexts; some SEN plausibly also have the form of: “If you’d like to find out about topic X, this is whom you ought to turn to for information, and this is whom you ought to ignore.” This would explain why agents form bad beliefs only regarding some topics, yet SEN would still play a role here. ii) As discussed in Sect. 3, SEN and motivated cognition plausibly interact in complex ways. For example, it might be that a SEN instructs agents to protect beliefs about a certain topic. Both of these points should be kept in mind when investigating this.

Second, we might find that agents *from the same social group* tend to form bad beliefs to different degrees. Again, seeing as the same SEN likely governs these agents, it would seem that other factors are explanatorily relevant. Let me again qualify this somewhat: Even though agents might be governed by the same SEN, they might differ in how able or motivated they are to conform to these norms. Thus, agents who tend to form more bad beliefs are those able and willing to conform to the norms, whereas others are less so. So, whilst qualified, bad SEN would still figure centrally in accounting for bad beliefs.

Ultimately, I think I can be fairly conciliatory about both of these points: What my view predicts is that there will be stable between-group differences in ES that are due to SEN. My view doesn’t predict that there won’t be other relevant explanatory factors. Estimating the strength of these different factors is a task for future empirical work. In conclusion, then, both of these points make clear, I think, that the role of SEN in ES and bad beliefs deserves closer empirical scrutiny—an insight that is due to the perspective gained from the ESSEN-framework.

## Social epistemic norms and blame

The framework also helps to capture the complex and often contextual normative landscape surrounding bad beliefs more adequately.

On Flores’ view, “epistemic styles” is a descriptive notion. But it seems intuitive that ES can be appropriate or inappropriate, rational or irrational, blameworthy or praiseworthy in different contexts. We can capture this by paying attention to the kind of normativity we get from the function that ES have: There’s an evaluative (and often, derivatively, a prescriptive) kind of normativity that comes with functions (see, e.g., Simion, 2019; Graham, 2019).^11^ On this view, an ES is good (or otherwise to be positively evaluated) as an ES if it fulfils its function of acquiring epistemic goods in the agent’s specific context. And so, an agent’s ES (and perhaps, by extension, that agent) can be negatively evaluated if their ES is *not* conducive to acquiring epistemic goods, given that agent’s specific context. It can be positively evaluated if it helps to (perhaps safely, reliably, or other epistemic qualifications) attain epistemic goods, given that agent’s specific context. For example, an ES that’s distrusting of state authority might be appropriate given the historical and political context in which an agent belonging to a marginalised group might find themselves in, e.g., if it’s about the kinds of political rights that are being granted by the state to that group. But such a style might be inappropriate if it’s employed by a privileged group member, given their specific context and history.

Following this, one might think that agents with bad beliefs are to be negatively evaluated as they employ an ES that doesn’t fulfil its function in their respective contexts. But what happens if we consider the insights from above, i.e., that SEN influence agents’ ES? The question then becomes whether agents should be positively or negatively evaluated for conforming to a SEN that leads to productive or unproductive ES.

I’ll tackle this question via the notion of epistemic blame. Roughly put, epistemically blameworthy believers violate the epistemic obligations that pertain to them. Epistemic obligations, in turn, are obligations whose fulfilment leads to achieving epistemic success or the promotion of epistemic goods (see, e.g., Feldman, 2002; Millar, 2021). For example, an agent might be blameworthy for forming their beliefs on incomplete evidence, as they failed to gather more evidence and thus didn’t fulfil the obligation of believing on adequate evidence.^12^ Who then is to be blamed for bad beliefs if they result from ES that SEN have misregulated?

Consider first that we might want to blame *groups*—rather than individuals—for regulation-failures of SEN. Which social norms are operative in a given community is something that’s at the remit of a collective rather than an individual. Social norms not only solve collective action problems, but they themselves are also a kind of collective undertaking—they require agents acting in concert to be upheld or changed (see, e.g., Bicchieri, 2016; Legros & Cislaghi, 2020). One might think that social groups are thus to be blamed for not structuring their epistemic environments in ways conducive to the attainment of epistemic goods.

We can draw from existing work to account for some cases of regulation failures due to cooperation-norms: These are cases where even though the norm’s content is legitimate, due to the lack or right kind of incentives, agents form or share their beliefs in ways not conducive to attaining epistemic goods. Here it seems that both groups and agents deserve some blame. Groups are to be blamed for employing insufficient SEN. But the individual in question also is to be blamed as they do not conform to their group's norms and the legitimate epistemic obligations encoded in them. As Palermos notes, in such cases, “epistemic collaborations fail or malfunction due to having some of their members’ acting ‘out of line’ by breaking joint commitments” (Palermos, 2022, p. 345), and it seems that they’re deserving of blame in virtue of doing so.^13^

There’s likely more to say on how groups should be blamed for their SEN and/or how individuals should be blamed for (not) conforming to them. But I’d like to draw our attention to another class of cases that have found less attention in the extant literature and will complicate how we think about the blameworthiness of people with bad beliefs. Here, agents don’t go against the operant SEN but rather conform to them. Agents behave as their group requires them to do, and it’s precisely because of this that they attain bad beliefs. In contrast to the cases above, agents conform to these norms for what plausibly are respectable reasons^14^: They’d like to coordinate or, more broadly, cooperate with others to attain epistemic goods. In consequence, it is much less clear what we should blame them for.

One option is the following: We might want to blame agents for not behaving in ways so that the operative social norms in their group would be changed. Agents might have violated their obligations to participate in a joint action—together with others in their group—to ensure that the SEN that guide them are reliable (or safe, or other epistemic qualifications). Blame here would either be shared between agents or ascribable to them because of their membership in a blameworthy social group (see, e.g., Millar 2021).^15^

Whilst this is intuitive, in the following, I argue that in these cases, there’s a good case to be made for:

(i) *some* agents being largely blameless (and others being blameworthy) and
(ii) more generally, agents' blameworthiness being subject to various contextual factors that render all-encompassing judgements of their epistemic conduct inappropriate.

To see this, consider that whether agents are to be blamed for conforming to or not aspiring to change the SEN of their groups will, at least in part, depend on whether we think they should be aware of the respective norm’s epistemic status. It’d be odd to blame an agent for something they weren’t required to have been aware of. These cases complicate our picture of the blameworthiness of people with bad beliefs because whether such agents should’ve been aware of their norm’s epistemic status is complex and context-specific.

First, notice that evaluating a norm regarding its reliability (or other qualifications) is exceedingly difficult. Consequently, requiring non-ideal agents to do this themselves demands too much of at least some of them, thus violating ought-implies-can principles (see Carr, 2022; McKenna, 2023).^16^

The reason for this is that social norms and the behaviours they regulate are opaque (see, e.g., Henrich, 2015; Sterelny, 2021, p. 39). It’s often unclear why conforming to them would be advantageous to agents and/or the promotion of which goods social norms are supposed to achieve (Bicchieri, 2016, p. 2). Additionally, even if we’d find out how social norms work—what function they have, how they fulfil it—it’d still be very difficult to evaluate them, just because the kinds of behaviours they regulate are complex, the product of contingent historical processes, and specific to particular contexts.

The same holds of SEN: They usually aren’t stated as “one ought to believe that p iff”, followed by some conditions that make their purpose obvious. On the contrary, it’s often unclear what the epistemic import and function of many social norms is—as is the case in, e.g., “Respect your elders!”, “Be loyal to your friends!”. In addition, even if we were clear on what precisely these norms are to achieve—and how they seek to achieve this—it’s still difficult to evaluate under which conditions they’re reliable. As the above discussion of the “Do your own research!”-norm illustrates, these norms manage complex, contradictory, and context-specific needs of communities and their members. The reasons for/against, e.g., believing with your friends, are equally complex and context-specific—these are topics philosophers are working on (see, e.g., Goldberg, 2022). Evaluating such norms is outside of the remit of individuals.

A lack of clear and direct feedback on any particular instance of norm-conformity further hinders awareness of a SEN’s reliability. Even if agents conform to a SEN, it often won’t be clear whether their belief was successful (i.e., knowledgeable) and how far this is due to their conforming to that norm. This is partly due to a lack of access—it won’t be clear to agents whether their belief does conform to a norm based on introspection—but also due to these agents’ epistemic environment being structured in ways so that counterevidence isn’t available to them (see, e.g., Nguyen, 2020).

As a result, we can see that many lay-believers—though there will be exceptions, especially amongst those with access to more resources—will not be in a position to be aware whether the norms they conform to are reliable.

One might agree with this result yet still think that agents should be aware that other social groups conform to different norms. Shouldn’t this decrease their confidence in their group’s SEN? Let me mention two things here, both point to *some* agents being reasonably distrustful of other groups’ social practices.

First, Meylan and Schmidt (2023), for example, describe why agents of marginalised social groups reasonably distrust medical experts because of systemic injustices within medical practice—and this likely generalises to other cases where marginalised people interact with “dominant” institutions. Levy (2021) describes how agents are sometimes reasonably sceptical of scientific experts more generally due to problems with how science is practised—e.g., the replication crisis and predatory journals. Carr (2022) describes that we should expect reasonable differences in social practices due to different needs, histories, and compositions groups might have. Additionally, certain norms might be more easily implemented or sensible in some contexts rather than in others. Agents might thus reasonably consider differences in norms to be due to such differences between their groups.

Second, as described above, evaluating social norms is exceedingly difficult. As such, it makes sense to outsource this to others. But whom should one defer to? Notice that which social norm one is supposed to conform to is a normative question. As Rini (2017) argues, it makes sense to trust agents who share one’s normative outlook regarding normative matters. Importantly, reasonable proxies for who shares one’s values—e.g., group membership—will likely coincide with the norms agents conform to. So, again, agents might reasonably defer to those who share their normative outlook and thus come to stick with their norms.

This shows that at least some agents do have reasons to be sceptical of other groups’ social practices. There are thus reasons to consider them blameless in conforming to the SEN of their group.

Lastly, notice that thinking of agents with bad beliefs in monolithic ways is likely unproductive. We shouldn’t think that their social identity is wholly confined to the outskirts of one group or that they’re part of a social environment entirely structured by bad SEN. Whilst that might be true for some agents, I’d suggest that the social identities and environments of a large subset of people with bad beliefs are manifold and complex. They’re a part of different social groups governed by different norms.

Such complex social identities matter as agents that are part of different social groups are likely to be subject to different norms in different contexts. These agents could—qua their multifaceted social identity—come to be aware that conforming to different SEN is a live option for them. Agents can, perhaps over time, appreciate that there’s a tension between the practices of the different social groups they belong to. They might realise that behaving in a norm-conforming way in one group is norm-violating in another. I take it that agents subject to such conflicting norms *could* come to be aware that different norms exist and would, in principle, be viable options for them.

Now, agents might come to distrust one group they’re part of precisely because their norms conflict with those of another group the agent is part of. But there’s a certain degree of agency in how agents will deal with this tension; there’s agency in how and why agents choose to conform to one group’s norms and not the others’. And it’s in virtue of this that some agents might deserve blame for their bad beliefs. Notably, the above reasons for being distrustful of the social practices of *other* groups don’t necessarily apply here, as these are groups that agents themselves are part of.

But notice too that just as there are agents with sufficiently multifaceted social identities, others won’t be as lucky. Agents more or even entirely encompassed by social groups structured by deleterious SEN won’t as easily become aware that there’d be other norms to conform to.

The picture that emerges is thus one where some agents subject to unfortunate circumstances will be blameless in forming bad beliefs, whereas others won’t be. More generally, their blameworthiness depends on the kinds of resources, abilities, and environments they have at their disposal to make sense of a norm’s reliability, the kinds of reasons available to them for (not) considering others’ social practices, and the make-up of their social identities and communities.

## Conclusion

I’ve established a framework that lets us consider bad beliefs to result from regulation failures of bad social epistemic norms. I’ve shown why there are good reasons to consider some agents with bad beliefs—but not others—to be blameless in forming such beliefs and have drawn attention to some of the contextual factors that should influence our judgements regarding their blameworthiness.

There is, of course, much that is left to do. On the one hand, future research should investigate the degree to which bad beliefs are due to the different kinds of SEN I’ve discussed. In particular, the framework should be tested regarding its predictions, the evidence that would speak against it, as discussed in Sects. 4.1 and 4.3, and how it interacts with accounts discussed in Sect. 3. If there’s some truth to what I’ve suggested, possible social norm-based interventions for bad beliefs should also be considered in more detail. Additionally, we should come to better understand how bad SEN emerge in the first place and how this is related to the prevalence of bad ES within a community. On the other hand, future research should more closely consider which normative notions help us to best make sense of the normativity of ES and how groups are to be blamed for their SEN. Lastly, as the blameworthiness of agents with bad beliefs is subject to the contextual factors I’ve outlined above, future research should strive to more precisely account for the actual contexts in which agents find themselves in.

## Footnotes

1 A worry one might have with the “epistemic parameter” terminology might be the following: Parameters should be measurable, yet it’s unclear how at least some of these parameters should or could be measured. For the purposes of this paper, I’ll keep using Flores’ terminology, though I acknowledge this worry that future work should address. My thanks to an anonymous reviewer for pointing this out.

2 Whilst this has certainly been a long-standing criticism of virtue epistemology (see, e.g., Fairweather & Alfano 2017), I remain neutral whether virtue-theorists truly do have to ascribe longstanding character traits to agents. My point here is simply that the notion of ES allows for context-sensitivity and that this makes the notion suitable for this paper’s explanatory aims.

3 Flores (2021, footnote 12) mentions that: Developing an account of the agency we have over our epistemic styles is beyond the scope of this [Flores’] paper “. I too will not give such an account, as my focus is different here.

4 Agents might also promote *epistemic* aims that are different from their social group’s (Mayo-Wilson et al., 2011).

5 My thanks to an anonymous reviewer for bringing this to my attention.

6 I’m saying «more or less widespread and systematic» since one might think that a certain number of bad beliefs are tolerable for the epistemic functioning of a social group. I tend to think that the amount of tolerable bad beliefs is less than what we might expect — epistemic ecosystems are fragile.

7 My thanks to an anonymous reviewer for bringing this to my attention.

8 Fraser’s account is also discussed as an example of ES in Flores (2021).

9 My thanks to an anonymous reviewer for this suggestion.

10 My thanks to an anonymous reviewer for this suggestion.

11 Making sense of the normativity of ES via functional normativity is, I think, a natural fit—not least because functional normativity has proven itself to be explanatorily powerful in epistemology and beyond. But I acknowledge that there might be other ways of making sense of ES’ normativity. One example might be Sosa’s (2007) virtue theory, as it’s similarly teleological in character.

12 There exist other ways of making sense of epistemic blame in the literature, e.g., via reasons instead of obligations. See Boult (2021) for an overview.

13 There’s an interesting question whether the kind of blame that conforming to certain SEN would merit would still be epistemic in nature. One might think it is, since the question of whether to conform to these norms is relevant to the attainment of epistemic goods. However, even if one disagrees on this point, as Palermos (2022) notes, we might, additionally or alternatively, hold them structurally or morally responsible for their behaviour.

14 As discussed in the section on IPC above, agents might also conform to these norms precisely because they allow them to protect cherished beliefs. This motivational difference is relevant to how we assess their blameworthiness. The cases I discuss assume a different motivation.

15 Naturally, agents might be differently blameworthy given their influence within the group. We’ve seen that high-status individuals might not be ideal instigators of change in the sense that they’re unlikely to act counter-normatively (see Sect. 4). But this doesn’t entail that they shouldn’t be blamed proportionally to the influence they’ve got within the group. These agents *could* play a large role in changing their group’s norms, and that’s what we should blame them for.

16 We might also want to say that such agents are excused or exempt from appreciating the reliability of norms, as this would be beyond their means (Littlejohn, 2015).
